# Patterns of antibiotic use for acute febrile illness in resource-limited settings: a multicenter study in DR Congo, Kenya and Uganda

**DOI:** 10.3389/fpubh.2026.1837179

**Published:** 2026-05-29

**Authors:** Luciana Lepore, Jeanette Dawa, Raymond Odinoh, Silvia Situma, Luke Nyakarahuka, Sheila Makiala, Carolyne Nasimiyu, Daniel Mukadi-Bamuleka, Hervé Viala, Christian Ifufa, Katharina Kreppel, Robert F. Breiman, Barnabas Bakamutumaho, Justin Masumu, Kariuki Njenga, Veerle Vanlerberghe

**Affiliations:** 1 Department of Public Health, Institute of Tropical Medicine, Antwerp, Belgium; 2 Afya Ushahidi Foundation, Nairobi, Kenya; 3 Washington State University Global Health Program, Nairobi, Kenya; 4 Center for Research in Emerging Infectious Diseases-East and Central Africa, Nairobi, Kenya; 5 Department of Animal Science, Pwani University, Kilifi, Kenya; 6 Department of Biosecurity, Ecosystems and Veterinary Public Health, College of Veterinary Medicine, Animal Resources, and Biosecurity, Makerere University, Kampala, Uganda; 7 Viral Hemorrhagic Fever Surveillance Program, Uganda Virus Research Institute, Entebbe, Uganda; 8 Department of Global Health, Rollins School of Public Health, Emory University, Atlanta, GA, United States; 9 Department of Virology, Institut National de Recherche Biomédicale, INRB, Kinshasa, Democratic Republic of Congo; 10 Department of Medical Biology, Faculty of Medicine, University of Kinshasa, Kinshasa, Democratic Republic of Congo; 11 Laboratoire Rodolphe Mérieux INRB-Goma, Goma, Democratic Republic of Congo; 12 Service of Microbiology, Department of Medical Biology, Kinshasa University Hospital, Faculty of Medicine, University of Kinshasa, Kinshasa, Democratic Republic of Congo; 13 Department of Epidemiology and Global Health, Institut National de Recherche Biomédicale, INRB, Kinshasa, Democratic Republic of Congo; 14 Infectious Diseases and Oncology Research Institute, University of the Witwatersrand, Johannesburg, South Africa; 15 Department of Arbovirology, Emerging and Re-emerging Infectious Diseases, Uganda Virus Research Institute, Entebbe, Uganda; 16 Cellule d'appui à la Recherche, Institut National de Recherche Biomédicale, INRB, Kinshasa, Democratic Republic of Congo; 17 Paul G. Allen School for Global Health, Washington State University, Pullman, WA, United States

**Keywords:** acute febrile illness, antibiotic, AWaRe, Democratic Republic of the Congo, East Africa, Kenya, Uganda, undifferentiated fever

## Abstract

**Introduction:**

In East Africa and beyond, uncertainty regarding the underlying aetiology of febrile illnesses may result in inappropriate treatment. In our study, we aimed at enhancing our understanding of antibiotic use patterns, prescribing practices and factors influencing antibiotic use in this region by exploring the pathways of care for acute febrile illnesses in three countries in East Africa.

**Methods:**

Between October 2021 and February 2024, acute febrile patients were enrolled in six health facilities in the eastern Democratic Republic of the Congo (DRC), Kenya, and Uganda. Our cross-sectional study assessed sociodemographic and clinical data along with initial diagnosis and antibiotic treatment record, collected at the initial outpatient and enrolment visit.

**Results:**

A total of 4,806 subjects were enrolled: 1,370 in DRC, 1,468 Kenya, 1,968 Uganda. About a quarter of the total population (23.3%) reported having already sought care before enrolment, most frequently in DRC (36.6%) at pharmacies (83.9%), and in Uganda (27.9%) at hospital (84.1%). Fifty percent of the DRC study population reported antibiotics use before enrolment, much higher than Kenya (3.3%) and Uganda (11.2%). Undifferentiated febrile illness was suspected in 37.5% of cases. At enrolment, in all countries, antibiotics were prescribed for 72.9% of cases (DRC 87.2% > Kenya 68.9% > Uganda 65.8%), with a non-negligible prescription of *watch* antibiotics (33.0%), prevalent in DRC (62.2%). In Kenya and Uganda, *access* antibiotics prevailed (80.9 and 67.1%, respectively). Prescription of *watch* antibiotics for undifferentiated febrile illnesses was strongly associated with a positive Widal test and access to hospital.

**Conclusion:**

Different patterns of access to care and antibiotic use for acute febrile illness were observed among countries. DRC had the highest level of pharmacy attendance prior to accessing the formal healthcare sector, as well as the highest reported use of antibiotics before formal care. It also showed the highest proportion of *watch* antibiotic use within the formal healthcare sector, even among patients without a confirmed diagnosis. Urgent targeted action is needed through effective and sustainable antimicrobial stewardship programs targeting both the formal and informal health sectors.

## 1 Introduction

Fever is one of the most common complaints among patients seeking healthcare services in low- and middle-income countries, where the burden of infectious diseases remains high and persistent (1). Sub-Saharan Africa (SSA) carries the heaviest load in terms of infectious diseases, with febrile illnesses accounting for an estimated 184 per 1,000 of hospital visits and over 16 million hospital admissions in 2014 (2, 3). The differential diagnosis of non-malaria febrile illness remains extremely broad, ranging from viral, bacterial, parasitic to fungal infections (4). In healthcare settings in SSA, with restricted availability of microbiological diagnostic tools (5), clinical history and physical examination are critical to determine the cause and focus of the infection (i.e., respiratory, gastrointestinal, or other) (6). However, some febrile illnesses present without organ-specific symptoms but with fairly non-specific initial complaints such as headache, chills and myalgia, and they are classified as undifferentiated fevers (4) whose aetiology often remains unknown (7, 8). Given the wide range of causes of fever with limited availability of diagnostic tools, and constraints in access to care and human resources in SSA, the diagnosis and management of febrile illnesses remain challenging, potentially leading to empirical treatment with inappropriate or inadequate antibiotics (9). It is worth emphasizing, moreover, that in SSA, self-medication practices are widespread, with over 50% of the population reporting self-medication with antibiotics bought in chemists/drug stores and pharmacies (10, 11) and that the increase in empirical antibiotic prescriptions has also coincided with a reduction in empirical treatment of malaria, thanks to the wider use of malaria rapid tests. While there is a well-established correlation between the development of antimicrobial resistance (AMR) and antibiotic overprescription (12), globally there has been a dramatic increase of over 65% in antibiotic use between 2000 and 2015 (13), resulting in AMR to be one of the major global health challenges, potentially larger than infectious diseases (i.e., HIV and malaria) (14). According to a recent global analysis based on predictive statistical models, AMR is estimated to have directly caused 1.27 million deaths and contributed to approximately 4.95 million (3.62–6.57) deaths worldwide in 2019, with the highest share in the western SSA region (15, 16). Worrying predictions estimated that by 2050, 10 million deaths per year could occur if the inappropriate use of antibiotics is not addressed (17).

In 2015, the World Health Organisation (WHO) developed a Global Action Plan to guide the strategy on AMR with the launch of the Global Antimicrobial Resistance Surveillance System (GLASS), a collaborative effort to standardize antimicrobial surveillance (18). The country-specific National Action Plans (NAPs) on AMR that followed, showed some variability between countries, with high-income countries being more prominently represented. However, Kenya’s NAP stood out as a positive case, demonstrating a comprehensive approach, supported by a governance framework operating at national and regional (county) levels through multisectoral government agencies (14). Furthermore, to monitor the antibiotic use alongside the implementation of antimicrobial management policies, the WHO introduced the AWaRe classification framework in 2017. This framework, aligned with the recommendations of the WHO Essential Medicines List and the Essential Medicines List for Children, groups antibiotics into four categories: *access* antibiotics in principle widely available as first, or second-choice, treatments for common infections, *watch* antibiotics which should be restricted for specific indications because of their higher risk of resistance selection, *reserve* antibiotics as last-resort antibiotics against multi-resistant organisms, and *not recommended* antibiotics (9, 19). The WHO proposal in the “Adopt AWaRe” campaign was to achieve, by 2023, at least 60% of national antibiotic consumption coming from the *access* group of antibiotics (9), updated to at least 70% by 2030 (20). A recent systematic review covering West African countries, previously identified as the main global hotspot for antibiotic resistance, reported high antibiotic consumption in the region, with 64% of *access* antibiotics, and the need for further implementation of antimicrobial stewardship programs, currently at an early stage and limited to tertiary and secondary hospitals (15).

In East Africa, where hospitals still have few accessible diagnostic tools with limited capacity to accurately identify multiple pathogens, undifferentiated fevers have been diagnosed in up to 64% of the adolescent and adult population, indicating that nearly two-thirds of the population seeking healthcare in East Africa may receive inadequate treatment due to the unknown aetiology of potentially life-threatening fever (1). The aim of our study was therefore to assess antibiotic use patterns, prescribing practices and factors influencing antibiotic use in this region by exploring the pathways of care for acute febrile illnesses in three countries in East Africa: in the DRC (eastern part), Kenya and Uganda. This was realized as part of a multi-country initiative of the Center for Research on Emerging Infectious Diseases - East and Central Africa (CREID-ECA).

## 2 Methods

### 2.1 Study design, settings and participants

This cross-sectional study used baseline enrolment data from a parent multicenter study to examine care seeking pathways and antibiotic use at first outpatient consultation. The parent study (prospective longitudinal) was conducted between October 2021 and February 2024 investigating the maintenance and transmission of Rift Valley fever (RVF) in East Africa during inter-epidemic periods. Acute febrile patients of ≥10 years presenting for an outpatient consultation at six health facilities, in eastern DRC (1 hospital), in central Kenya (1 hospital and 1 dispensary), and in southwestern Uganda (1 hospital and 2 health centers), were prospectively enrolled. Consenting subjects with a history of fever (body temperature ≥37.5 °C) in the last 4 weeks and/or unexplained bleeding and/or unknown infectious illness treated for >7 days and unresponsive to treatment, were enrolled. Cases diagnosed with malaria were included up to a maximum of 20% of the study sample, the number of malaria cases enrolled per day was capped and continuously monitored throughout the study period. Pregnant women were included due to their potential RVF exposure and the RVF-associated abortive effects observed in livestock, which remain poorly characterized in humans (21). We excluded non-consenting subjects and patients with specific and already diagnosed illness, such as urinary tract infection or COVID-19, and patients hospitalized for more than 48 h in the past 14 days. The sample size over the 2 years was estimated to 1600 participants for the single study site in the DRC and 707 participants per study site in Kenya and Uganda. The sample size was calculated to detect a seasonal difference of RVF acute cases, estimated at 3% in the rainy season and 1% in the dry season, with a confidence level of 95%, a power of 80% and a precision of 2% (22, 23).

### 2.2 Data collection and management

All field data were de-identified and collected by electronic questionnaires on REDCap (24). Data quality control was carried out throughout the study, ensuring the cleaning and harmonization of data collected in different sites and from different countries. Anonymized data from the six study sites were merged, resulting in a single study database. Relevant data were cleaned and prepared for analysis.

Study data were collected at the time of the initial outpatient visit, coinciding with study enrolment, by the healthcare provider/study staff, supported by clinical records. These included socio-demographic and clinical data, such as clinical signs recorded on physical examination and symptoms reported by the patient, laboratory investigations, initial working diagnosis assigned by the healthcare provider, initial management and treatment decision including antibiotic prescription. Patients were also asked whether and where they had sought previous care, and to list and name any medicines or treatments they had already received during the current fever episode. The clinical data collection form was adapted from the Febrile Illness in Kinshasa and Kimpese_FIKI2 Study (ClinicalTrials.gov NCT04760678) running concurrently in different locations in DRC. Given that follow-up visits were designed to collect data aligned with the primary objectives of the parent study on RVF, and to avoid bias from loss to follow-up, our cross-sectional analysis was limited to data collected at enrolment.

**Figure 1:**
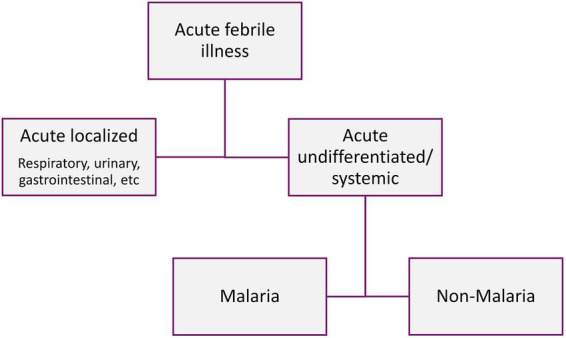
Working diagnosis flowchart at enrolment. Conceptual framework based on Bhargava et al. (4).

Among socio-demographic data, the level of education was defined as low schooling until completion of primary school and high schooling for levels beyond primary school completion. Among occupations, skilled ones were defined as those requiring formal education or technical training (e.g., engineers, teachers). All other occupations were classified as unskilled. Healthcare workers (HCWs) and farmers were considered separately. For initial laboratory testing, malaria tests performed on-site in accordance with the guidelines of the respective countries’ Ministry of Health were recorded. These included rapid diagnostic testing (RDT) to detect the parasite lactate dehydrogenase (pLDH) or histidine-rich protein 2 (HRP2) RDT and/or a thick blood smear. The Widal agglutination test, a serological test for the detection of *Salmonella* typhi and paratyphi antibodies, was also recorded when requested by the healthcare provider based on the patient’s clinical presentation. However, as highlighted in the WHO AWaRe antibiotic book, Widal serology is not a reliable method for diagnosing acute enteric fever, as a positive result may reflect prior infection rather than active disease (19). For the determination of the initial working diagnosis, the classification from Bhargava *et al*. (4) was adapted, distinguishing acute febrile illness in acute localised infection, with organ involvement, and acute undifferentiated febrile illness, including systemic illness, in the absence of specific organ involvement (Figure 1). More than one initial working diagnosis could be recorded at enrolment based on patients’ signs and symptoms and the clinical assessment, while non-malaria undifferentiated febrile illness was mutually exclusive with other diagnostic categories. If organ involvement was not specified or initial working diagnosis absent or unknown, the case was categorized as undifferentiated febrile illness.

Antibiotics prescribed at enrolment were classified into *access/watch/reserve* categories based on the WHO AWaRe classification (19). Anti-tuberculosis treatment and topical antibiotics were not included in this classification. In the case of patients receiving more than one antibiotic belonging to different categories, the most critical category was assigned (*reserve > watch > access*). The observed prescribing patterns at the facility level were evaluated based on the “Adopt AWaRe” campaign targets as indicative proxies - not strict performance measures–as these targets are not designed for facility-level assessment.

### 2.3 Data analysis

Data were analyzed using R statistical software version 4.4.1 (25). Results were summarized using descriptive statistics with numerical variables expressed as median and interquartile range (IQR), where appropriate, and categorical variables as percentages. Logistic regression models were fitted with reported antibiotic use prior to enrolment (country-specific) and *watch* antibiotic prescription at enrolment (undifferentiated febrile illness syndrome of the overall study population) as the main outcome variables. The chi-square test and Fisher’s exact test were used to compare proportions, with a significance level of 5%. Univariate analysis (crude odds ratios) was performed to assess the association between each independent variable and the outcome of interest. Variables with *p* < 0.1 were selected for multivariate analysis using a manual elimination process. Adjusted odds ratios (aOR) and 95% confidence intervals (CI) were calculated and reported, considering a *p*-value < 0.05 as statistically significant. Given the low proportion of missing values (<5%) and minimal potential for bias, a complete case analysis was performed. Potential interactions between key predictor variables were assessed.

## 3 Results

### 3.1 Overview of the study population

A total of 4,806 subjects were enrolled over the overall 2-year period: 1,370 (28.5%) from the DRC, 1,468 (30.6%) from Kenya and 1,968 (40.9%) from Uganda. Study participants were recruited from first line hospital in DRC (all), while in Kenya and Uganda, recruitment through dispensaries (50.4%) and health centers (65.4%) prevailed respectively, compared to hospital for the remaining participants. Women represented 57.5% of the study population, and the overall mean age was 31 years (IQR: 22–44), slightly older in Kenya (mean age 35, IQR: 24–47) compared to DRC (mean age 29, IQR: 23–41) and Uganda (mean age 30, IQR 22–41). Unemployment prevailed in DRC (43.1%), while participants from Kenya and Uganda were mainly involved in farming activities (30.6 and 64.5%, respectively), both as crop and livestock farmers. Lower level of schooling was reported by 63.4% of the overall study population, specifically being 62.2, 74.4, and 48.8% in Kenya, Uganda and DRC, respectively.

**Figure 2:**
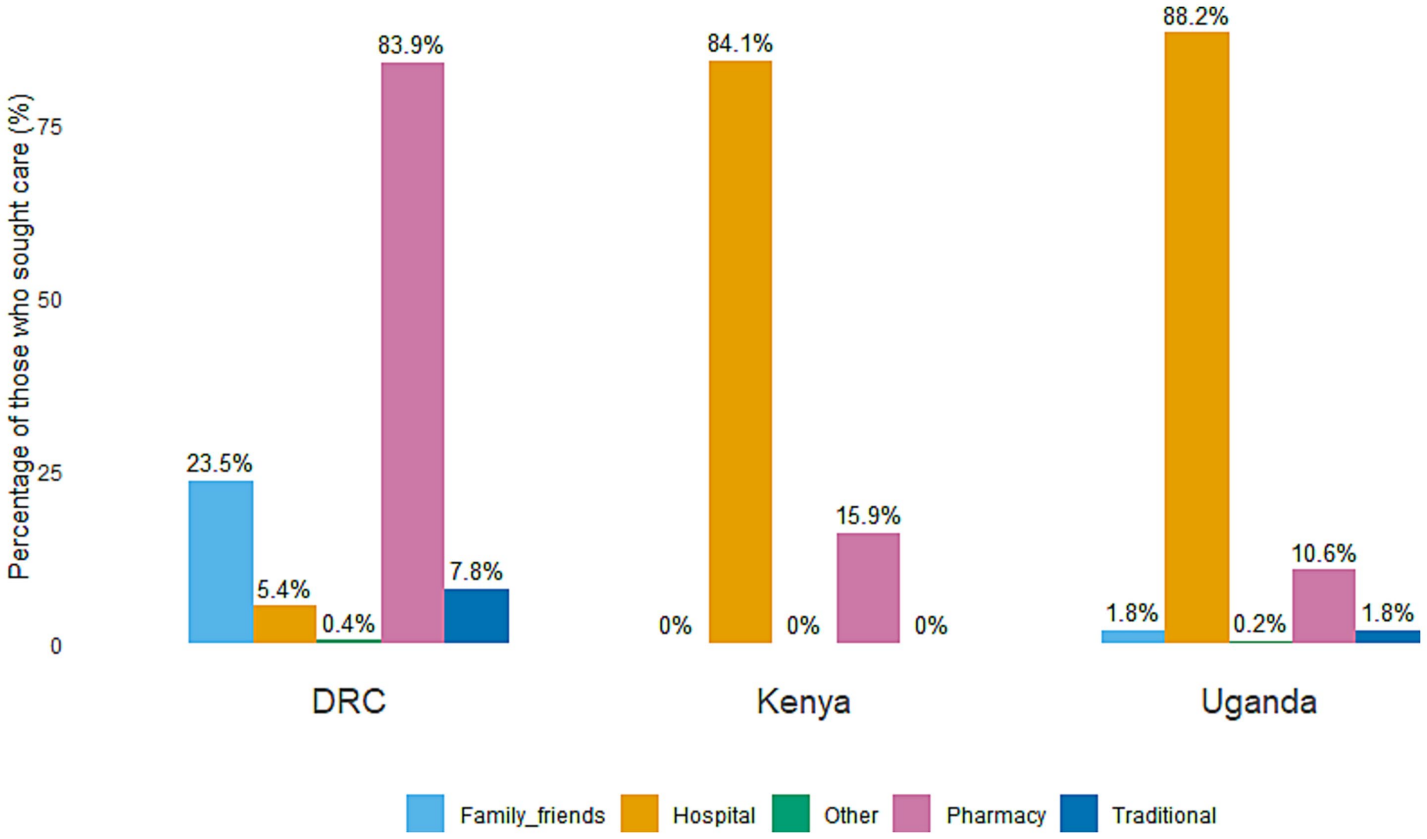
Type of care accessed by care-seekers prior to enrolment in the Democratic Republic of the Congo (DRC) (*n* = 502, 36.6%), Kenya (*n* = 69, 4.7%), and Uganda (*n* = 549, 27.9%), 2021–2024.

About a quarter of the total population (23.3%) reported having already sought care before enrolment: more frequently reported by participants from DRC (36.6%) and Uganda (27.9%), compared to Kenya (4.7%). Among these, access to pharmacy and chemist/drug store was most frequently reported in DRC (83.9%), while in Kenya and Uganda health facilities were preferred (84.1 and 88.2%, respectively) (Figure 2). Before enrolment, treatment with traditional remedies was reported predominantly by participants from Uganda (26.5%), while previous antibiotic therapy was 50.0, 3.3 and 11.2%, in DRC, Kenya and Uganda, respectively. Approximately 3% of participants from DRC and Uganda reported admission to a health facility prior to enrolment.

### 3.2 Clinical features and initial working diagnoses

About 95% of study population reported having had fever, the main study inclusion criteria, with onset within the week prior to inclusion (90.9%). Besides fever, other symptoms were reported by 99.3%, most frequently central nervous system symptoms (85.6%) including headache, and general symptoms (75.4%) including fatigue; less commonly musculoskeletal (59.3%), gastrointestinal (55.5%), respiratory (50.3%), and genitourinary symptoms (13.9%).

**Figure 3:**
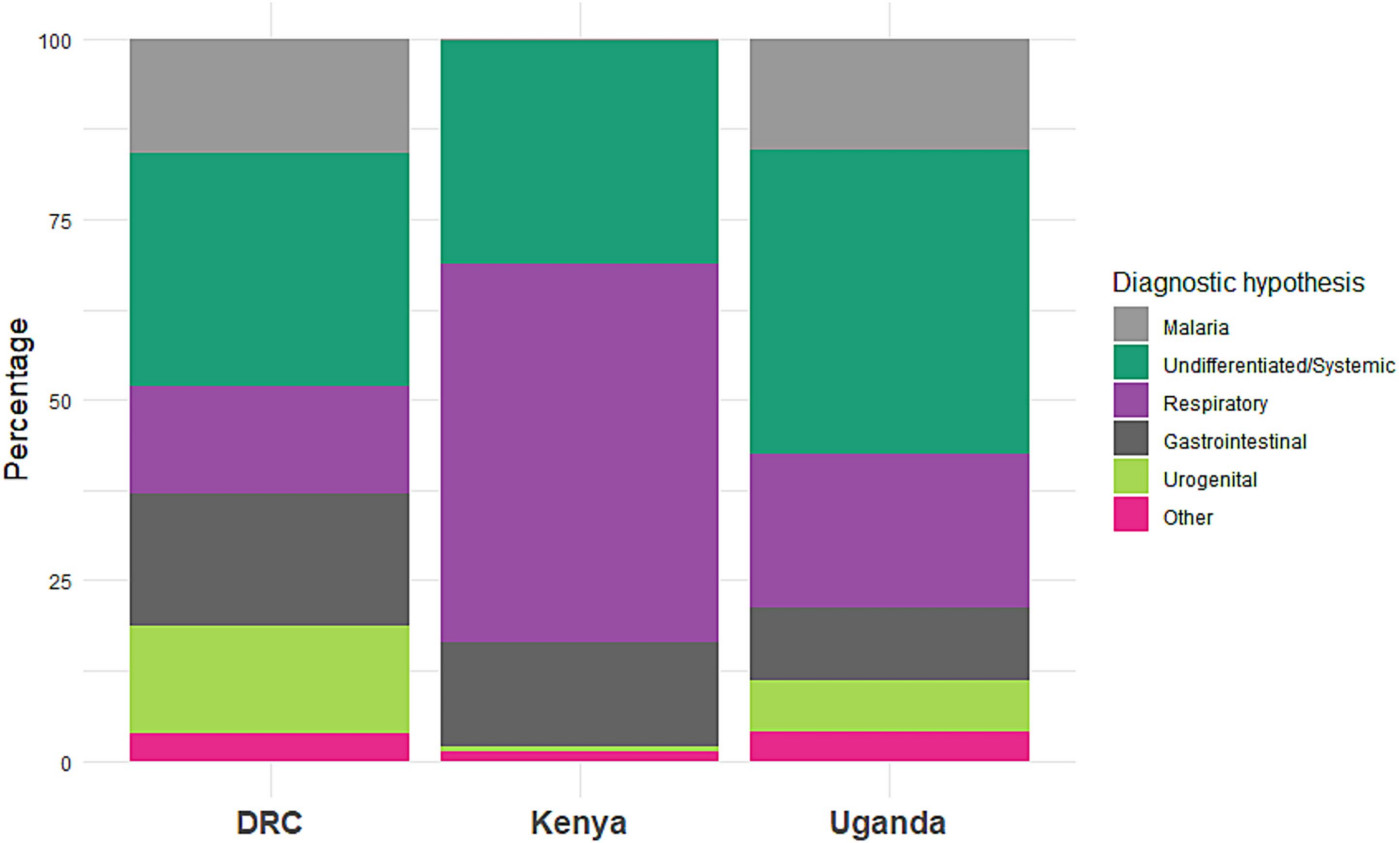
Distribution of initial working diagnosis at enrolment in the Democratic Republic of the Congo (DRC) (*n* = 1370), Kenya (*n* = 1468), and Uganda (*n* = 1968), 2021–2024.

At enrolment, the initial working diagnosis included undifferentiated febrile illness in 37.5%, respiratory in 29.9%, gastrointestinal in 14.2%, and urogenital syndromes in 7.9% of cases. The distribution of the initial working diagnosis at country level (Figure 3) was: undifferentiated febrile illness (excluding malaria) most prevalent in DRC and Uganda (34.5 and 44.2%, respectively), followed by gastrointestinal for DRC (19.3%) and respiratory for Uganda (22.4%). In Kenya, respiratory infections prevailed with 53.1%. DRC and Uganda recorded each 16% of malaria cases. Of note, the number of malaria case enrolments was set at a maximum of 20% of total enrolments per study protocol. Among undifferentiated illness cases in the DRC, nearly all were classified as suspected typhoid/paratyphoid fever (*n* = 461, 97.7%), based on clinical presentation and a positive Widal test, which however did not allow for a definitive diagnosis. In the other countries, bacteriemia was suspected in Kenya (*n* = 75, 16.3%), and bacteremia and sepsis in Uganda (*n* = 110, 12.6% and *n* = 273, 31.4%, respectively). As initial clinical management, most patients were administered antipyretics (89.7%) and antibiotics (72.9%), while only a minority of patients did not receive medical therapy (3.1%), 11.4% received malaria treatment. Hospitalization was deemed necessary in 8.1% of the study population.

Table 1 displays the study population key characteristics by country, according to the initial working diagnosis. Further sociodemographic characteristics and pre-enrolment data are provided in Supplementary Table 1. Malaria primarily affected younger populations, who had often sought care before enrolment in DRC and Kenya and were more frequently hospitalized at enrolment. Among symptoms besides fever, the triad of chills, headache, and arthromyalgia was most commonly detected in malaria cases, and fatigue, headache, and arthromyalgia in undifferentiated illness cases. Bleeding signs, reported in 9.1% of the overall population and particularly in Uganda (18.7%), were most commonly reported among patients with suspected undifferentiated illness. At least one of the malaria tests was positive in <10% of other working diagnoses than malaria. Widal tests were mainly performed in DRC showing predominantly positive results in cases classified as undifferentiated illness (59.5%), gastrointestinal illness (45.8%) and malaria (38.5%).

**Table 1:** Key characteristics of enrolled patients, by main initial working diagnosis, in the Democratic Republic of the Congo (*n* = 1370), Kenya (*n* = 1468) and Uganda (*n* = 1968), 2021–2024.

**Variable**	**DRC**					**Kenya**					**Uganda**				
	**Malaria**	**Undiff**	**Respir**	**Gastro**	**Urogen**	**Malaria**	**Undiff**	**Respir**	**Gastro**	**Urogen**	**Malaria**	**Undiff**	**Respir**	**Gastro**	**Urogen**
	**n 231**	**n 472**	**n 217**	**n 264**	**n 219**	**n 5**	**n 460**	**n 779**	**n 215**	**n 12**	**n 318**	**n 870**	**n 440**	**n 205**	**n 149**
Gender, female	146 (63.2)	309 (65.5)	138 (63.6)	180 (68.2)	185 (84.5)	1 (20.0)	246 (53.5)	363 (47.0)	116 (53.9)	10 (83.3)	136 (42.8)	524 (60.2)	225 (51.1)	109 (53.2)	111 (74.5)
Age, median (Q1-Q3)	29 (22–40)	32 (24–45)	27 (23–40)	29 (23–41)	29 (23–43)	25 (25–34)	37 (25–49)	34 (24–45)	35 (23–48)	46 (36–57)	24 (20–36)	31 (22–43)	29 (22–40)	30 (22–41)	30 (23–43)
Symptoms															
Reported fever	226 (97.8)	463 (98.1)	209 (96.3)	257 (97.3)	211 (96.3)	5 (100)	425 (92.4)	750 (96.3)	206 (95.81)	10 (83.3)	314 (98.7)	778 (89.4)	414 (94.1)	185 (90.2)	124 (83.2)
Fever onset ≤7 d	212 (91.8)	419 (88.8)	193 (88.9)	207 (78.4)	179 (81.7)	1 (20.0)	257 (55.9)	352 (45.2)	135 (62.8)	8 (66.7)	261 (82.1)	585 (67.2)	384 (87.3)	169 (82.4)	107 (71.8)
Fever at inclusion	182 (78.8)	373 (79.0)	188 (86.6)	181 (68.6)	163 (74.4)	5 (100)	126 (27.4)	167 (21.4)	41 (19.1)	2 (16.7)	272 (85.5)	590 (67.8)	366 (83.2)	174 (84.9)	109 (73.1)
Chills	158 (68.4)	132 (28.0)	43 (19.8)	48 (18.2)	46 (21.0)	5 (100)	193 (42.0)	549 (70.5)	70 (32.6)	1 (8.3)	270 (84.9)	613 (70.5)	274 (62.3)	138 (67.3)	81 (54.4)
Fatigue	138 (59.7)	282 (59.7)	134 (61.7)	144 (54.5)	93 (42.5)	5 (100)	178 (38.7)	397 (51.0)	66 (30.7)	0 (0)	200 (62.9)	639 (73.4)	215 (48.9)	117 (57.1)	77 (51.7)
Headache	221 (95.7)	438 (92.8)	197 (90.8)	223 (84.5)	185 (84.5)	5 (100)	302 (65.6)	623 (80.0)	107 (49.8)	0 (0)	299 (94.0)	743 (85.4)	391 (88.9)	178 (86.8)	105 (70.5)
Dizziness	106 (45.9)	158 (33.5)	55 (25.3)	79 (29.9)	43 (19.6)	0 (0)	53 (11.5)	29 (3.7)	16 (7.4)	0 (0)	195 (61.3)	566 (65.1)	111 (25.2)	68 (33.2)	45 (30.2)
Cough	40 (17.3)	153 (32.4)	159 (73.3)	41 (15.5)	33 (15.1)	1 (20.0)	95 (20.6)	627 (80.5)	24 (11.2)	1 (8.3)	122 (38.4)	347 (39.9)	336 (76.4)	44 (21.5)	32 (21.5)
Arhtomyalgia	144 (62.3)	264 (55.9)	109 (50.2)	89 (33.7)	64 (29.2)	4 (80.0)	191 (41.5)	369 (47.4)	59 (27.4)	0 (0)	260 (81.8)	665 (76.4)	311 (70.7)	138 (67.3)	83 (55.7)
Bleeding	2 (0.9)	26 (5.5)	3 (1.4)	5 (1.9)	6 (2.7)	0 (0)	15 (3.3)	8 (1.0)	5 (2.3)	0 (0)	33 (10.4)	223 (25.6)	44 (10.0)	34 (16.6)	32 (21.5)
Relevant conditions	24 (10.4)	40 (8.5)	17 (7.8)	8 (3.0)	11 (5.0)	0 (0)	14 (3.0)	26 (3.3)	7 (3.3)	2 (16.7)	17 (5.5)	42 (4.8)	11 (2.5)	10 (4.9)	5 (3.34)
Testing															
Malaria positive	227 (98.3)	1 (0.2)	7 (3.2)	21 (7.9)	10 (4.6)	5 (100)	1 (0.2)	0 (0)	1 (0.5)	0 (0)	310 (97.5)	1 (0.1)	18 (4.1)	5 (2.4)	10 (6.7)
Widal positive	89 (38.5)	281 (59.5)	23 (10.6)	121 (45.8)	61 (27.8)	0 (0)	0 (0)	0 (0)	0 (0)	0 (0)	1 (0.3)	4 (0.5)	0 (0)	1 (0.5)	1 (0.7)
Hospitalisation	66 (28.6)	61 (12.9)	15 (6.9)	42 (15.9)	19 (8.7)	0 (0)	0 (0)	0 (0)	0 (0)	1 (8.3)	68 (21.4)	64 (7.4)	26 (5.9)	30 (14.6)	10 (6.7)

d, days; DRC, Democratic Republic of the Congo; Gastro, gastrointestinal; n, number; Respir, respiratory; Undiff, undifferentiated; Urogen, urogenital.

### 3.3 Antibiotic use

**Figure 4:**
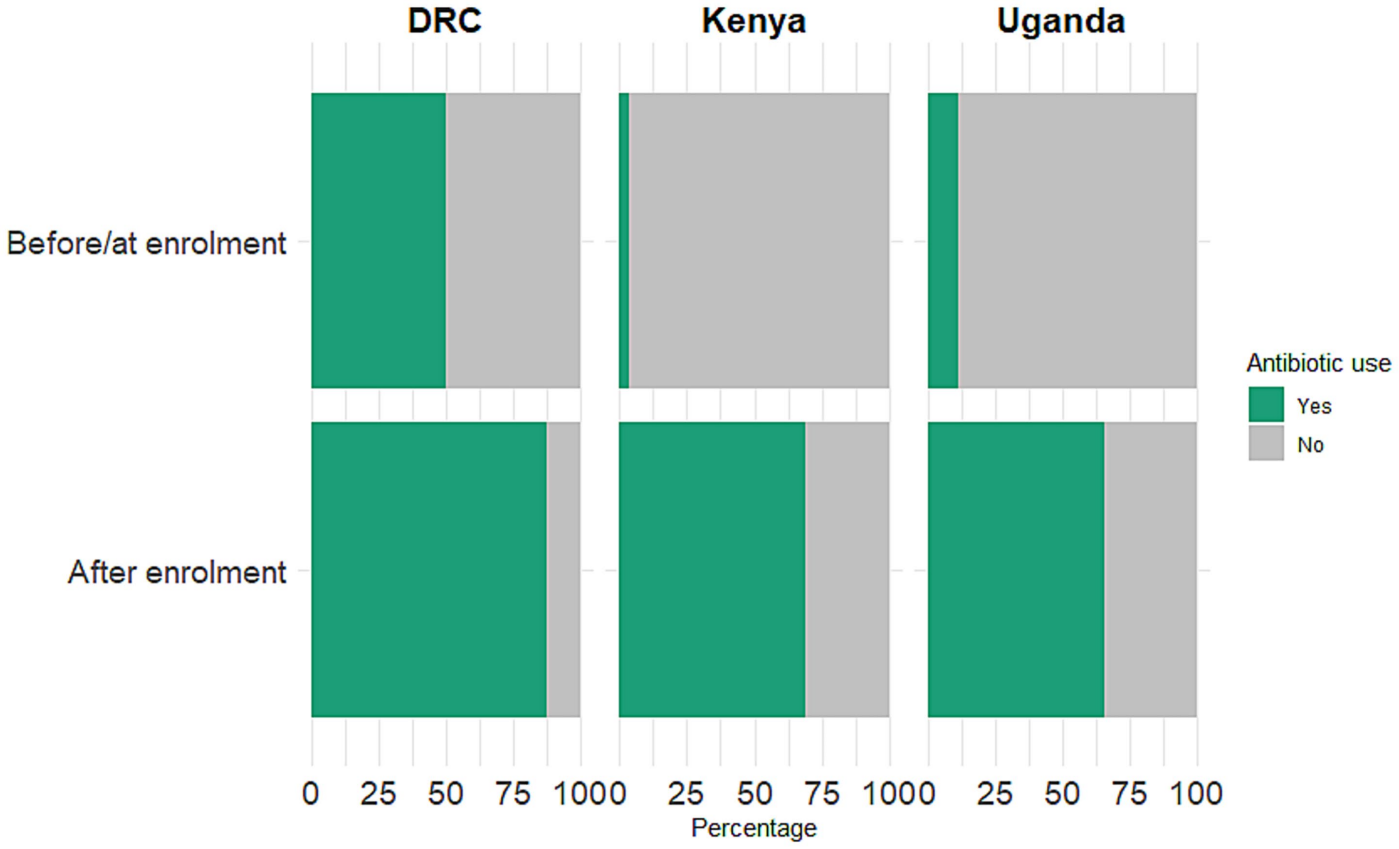
Antibiotic use prior and at enrolment, in the Democratic Republic of the Congo (DRC) (*n* = 1370), Kenya (*n* = 1468), and Uganda (*n* = 1968), 2021–2024.

The use of medical therapy, and in particular antibiotic therapy, was recorded, both in the period prior to enrolment and as part of the initial clinical management in the study health facilities (Figure 4). Prior to enrolment, antibiotic use was very different across countries, with DRC showing disproportionately higher reported use (50.0%) compared to Uganda (11.2%) and Kenya (3.3%). After enrolment, differences between countries narrowed and overall antibiotic use increased: however, the initial pattern was maintained, with the DRC prescribing the highest percentage of antibiotics (87.2%) compared to Kenya (68.9%) and Uganda (65.8%).

#### 3.3.1 Reported antibiotic use prior to enrolment

**Table 2:** Factors associated with reported antibiotic use before enrolment, in the Democratic Republic of the Congo (*n* = 1359), Kenya (*n* = 1416) and Uganda (*n* = 1878), 2021–2024.

Country	**Variable**	**cOR**	**95% CI**	***p*-value**	**aOR**	**95% CI**	***p*-value**
DRC	Occupation			<0.001			
	Farmer	ref			ref		
	HCW	2.15	1.05–4.59		2.44	1.12–5.52	
	None	3.27	1.77–6.36		3.35	1.72–6.92	**0.003**
	Skilled	3.11	1.60–6.34		3.43	1.66–7.46	
	Student	2.58	1.36–5.16		2.91	1.45–6.17	**0.004**
	Unskilled	2.16	1.13–4.34		2.01	0.99–4.27	0.06
	Trad med			<0.001			
	Yes	5.93	3.74–9.85		6.16	3.66–10.96	**<0.001**
	Prior admiss			0.004			
	Yes	2.81	1.44–5.91		3.22	1.49–7.47	**0.004**
	Seek care						
	Pharmacy	1.75	1.38–2.21	<0.001	2.14	1.64–2.82	**<0.001**
	Family/friends	0.14	0.08–0.23	<0.001	0.08	0.04–0.14	**<0.001**
	Symptoms						
	CNS	2.21	1.46–3.41	<0.001	2.20	1.42–3.49	**<0.001**
	Gastrointestinal	1.31	1.05–1.62	0.01	1.27	1.01–1.61	**0.045**
Kenya	Seek care						
	Hospital	128.6	62.94–276.28	<0.001	240.50	104.92–614.35	**<0.001**
	Pharmacy	58.22	16.92–229.66	<0.001	293.34	74.78–1330.71	**<0.001**
Uganda	Age group			0.015			
	10–20 yo	ref			ref		
	21–40 yo	1.57	1.05–2.41		1.81	1.06–3.15	**0.03**
	Above 40	1.91	1.22–3.04		2.10	1.15–3.91	**0.02**
	Occupation			<0.001			
	Farmer	ref			ref		
	HCW	2.49	1.29–4.52		3.06	1.41–6.44	**0.004**
	None	0.37	0.11–0.90		0.61	0.17–1.71	0.4
	Skilled	2.62	1.63–4.11		3.74	2.09–6.61	**<0.011**
	Student	1.03	0.64–1.59		1.98	1.07–3.62	**0.03**
	Unskilled	1.11	0.62–1.88		1.31	0.67–2.43	0.4
	Trad med						
	Yes	3.54	2.64–4.76	<0.001	2.10	1.48–2.97	**<0.001**
	Seek care						
	Hospital	15.04	10.78–21.32	<0.001	15.5	10.73–22.82	**<0.001**
	Pharmacy	2.01	0.97–3.80	0.044	5.11	2.25–10.81	**<0.001**

aOR, adjusted Odds Ratio; CNS, Central Nervous System; cOR, crude Odds Ratio; DRC, Democratic Republic of the Congo; HCW, healthcare worker; n, number; Trad med, traditional medicine; yo, years old; 95% CI, 95% Confidence Interval.

In the DRC study population, prior use of traditional treatments was strongly associated with antibiotic use (aOR 6.16, 95% CI 3.66–10.96). Other associated factors were: being unemployed (aOR 3.35, 95% CI 1.72–6.92) or a student (aOR 2.91, 95% CI 1.45–6.17), having previously been admitted to a healthcare facility (aOR 3.22, 95% CI 1.49–7.47) or having sought care at a pharmacy (aOR 2.14, 95% CI 1.64–2.82). Conversely, seeking treatment and advice from family and friends was associated with a substantial odds reduction (aOR 0.08, 95% CI 0.04–0.14). In Kenya, having sought previous care at a health facility or pharmacy were strong significant predictors aORs (>200), however with wide confidence intervals, likely due to the small sample size. Similarly, in Uganda, having sought previous care at a health facility and at a pharmacy were among the factors that showed the strongest association (15 and 5-fold increase, respectively), being an adult (age group 21–40 years aOR 1.81, 95% CI 1.06–3.15, and age group above 40 years aOR 2.10, 95% CI 1.15–3.91), HCWs (aOR 3.06, 95% CI 1.41–6.44) and participants involved in skilled occupations (aOR 3.74 95% CI 2.09–6.61). In the multivariate analysis, no association was found with either gender or educational level in any of the three countries. (Table 2 and Supplementary Table 3)

#### 3.3.2 Antibiotic prescription after enrolment

**Table 4:** Factors associated with *watch* antibiotic prescription for undifferentiated febrile illness, at enrolment, in the Democratic Republic of the Congo (*n* = 472), Kenya (*n* = 460) and Uganda (*n* = 870), 2021–2024.

**Variable**		**cOR (95% CI)**	***p*-value**	**aOR (95% CI)**	***p*-value**
Country	Kenya	ref	<0.001	ref	
	**Uganda**	3.04 (2.23–4.20)		1.77 (1.14–2.78)	**0.012**
	DRC	18.58 (13.26–26.43)		1.30 (0.81–2.12)	0.3
Enrolling facility	Dispensary	ref	<0.001	ref	
	Health cent	9.13 (4.98–18.77)		1.92 (0.86–4.58)	0.12
	**Hospital**	34.73 (19.22–70.74)		5.56 (2.74–12.31)	**<0.001**
Fever*	Yes	3.12 (2.52–3.87)	<0.001	1.84 (1.39–2.44)	**<0.001**
Symptoms	**General**	2.01 (1.58–2.59)	<0.001	1.96 (1.32–2.95)	**0.001**
	**Urogenital**	1.32 (0.99–1.75)	0.055	1.69 (1.21–2.37)	**0.002**
	**Muscokeletal**	1.48 (1.20–1.82)	<0.001	1.52 (1.12–2.06)	**0.007**
Widal positive	Yes	53.11 (32.29–94.40)	<0.001	37.86 (21.01–72.61)	**<0.001**
Admission	Yes	3.17 (2.19–4.66)	<0.001	2.52 (1.62–3.95)	**<0.001**

aOR, adjusted Odds Ratio; cOR, crude Odds Ratio; DRC, Democratic Republic of the Congo; n, number; * fever at enrolment, 95% CI, 95% Confidence Interval. Bold values and terms indicate statistically significant results (*p* < 0.05).

In DRC, antibiotic were prescribed in ≥ 80% of all initial working diagnosis, including malaria cases, in contrast to Kenya and Uganda, where ≤ 20% of malaria cases received antibiotics. In DRC and Uganda, antibiotics were most commonly prescribed for urogenital syndromes, while in Kenya antibiotic prescriptions prevailed for gastrointestinal syndromes, followed by respiratory ones. For undifferentiated illness, antibiotic prescriptions were limited to 40.2% of cases in Kenya, 90% in DRC and 70% in Uganda. About 13% of the overall population had received a combination of antibiotics, with a higher prevalence in the Ugandan study population (19.3%), especially for urogenital syndromes and gastrointestinal ones. Also in DRC and Kenya, the combination of antibiotics prevailed in these two syndromes, albeit to a lesser extent (Supplementary Table 2).

**Figure 5:**
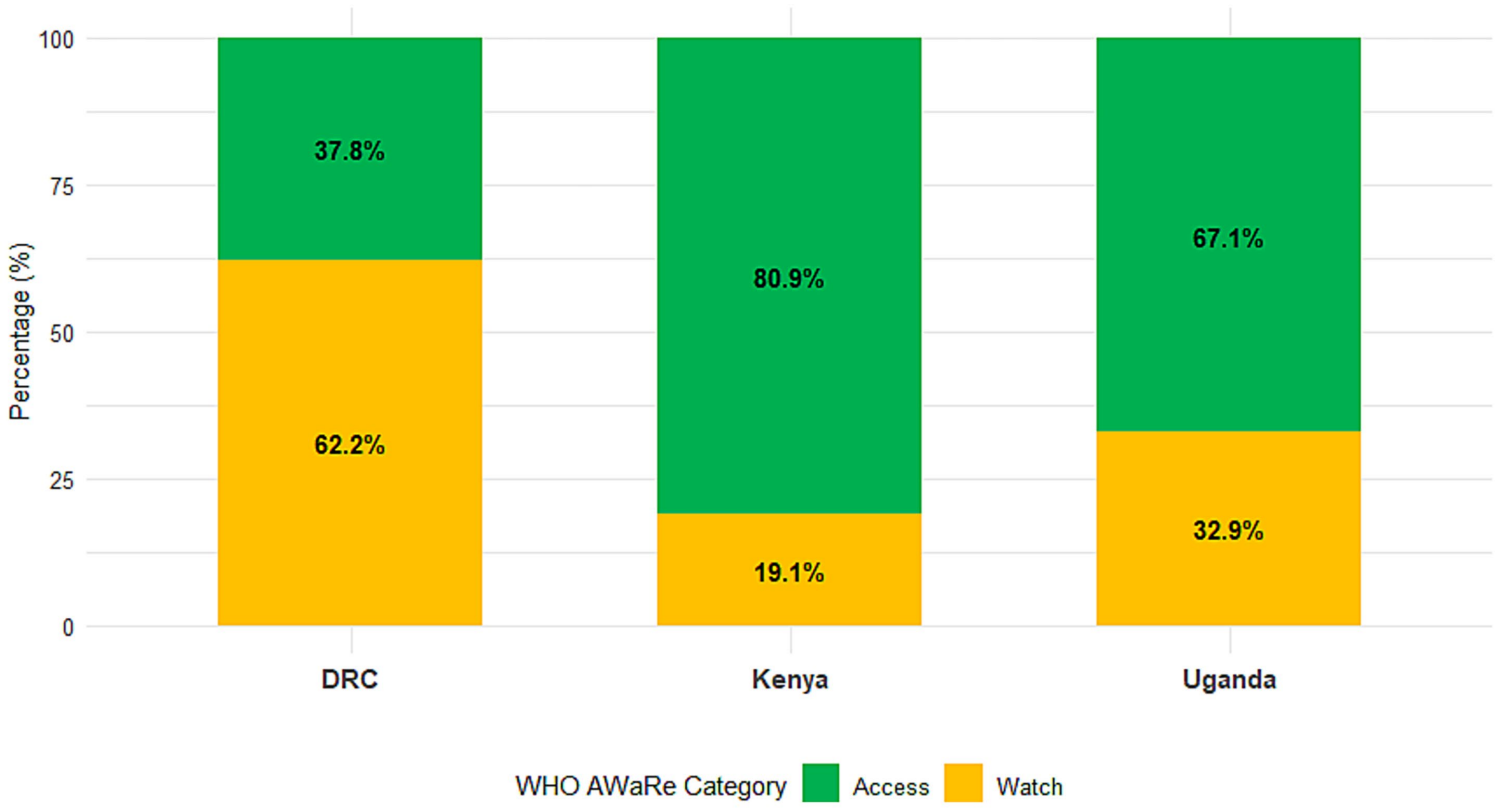
Distribution of *access* and *watch* antibiotics prescribed at enrolment, in the Democratic Republic of the Congo (DRC) (*n* = 1388 antibiotic prescriptions), Kenya (*n* = 1075 antibiotic prescriptions), and Uganda (*n* = 1703 antibiotic prescriptions), 2021–2024.

Cross-country differences in terms of antibiotic prescriptions were also observed regarding the antibiotic type, based on the WHO AWaRe classification (18). Out of the total number of prescribed antibiotics, DRC exhibited a marked use of *watch* antibiotics (62.2%). In contrast, the *access* group of antibiotics were the most prevalent in Uganda (67.1%) and Kenya (80.9%). No prescription of reserved antibiotics was found in any of the three countries (Figure 5).

**Figure 6:**
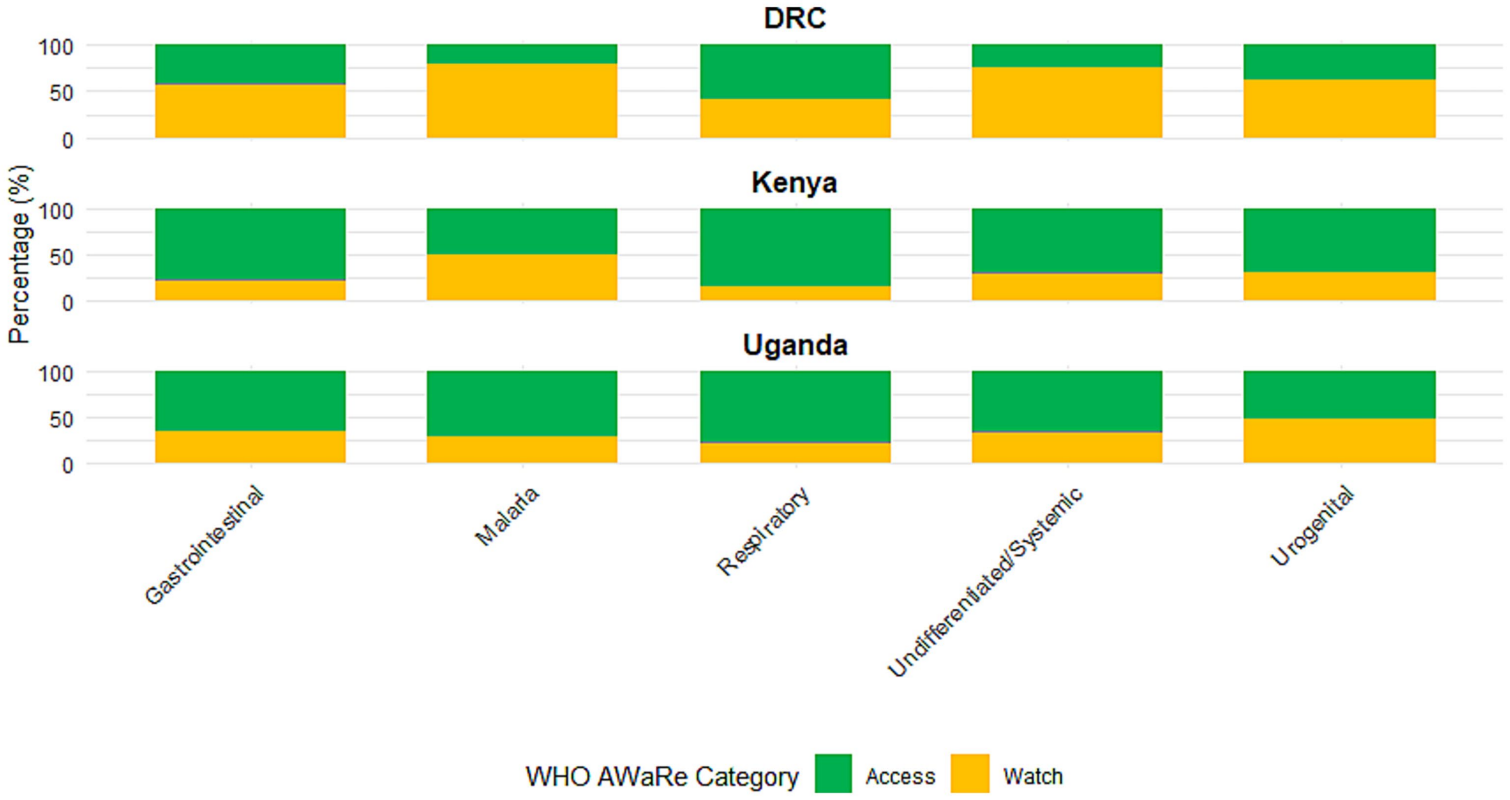
Distribution of *access* and *watch* antibiotics prescribed after enrolment stratified by main initial working diagnosis, in the Democratic Republic of the Congo (DRC) (*n* = 1388 antibiotic prescriptions), Kenya (*n* = 1075 antibiotic prescriptions), and Uganda (*n* = 1703 antibiotic prescriptions), 2021–2024.

Across all syndromes, *watch* antibiotics predominated in DRC, with the only exception being respiratory syndromes (Figure 6). Of note, *watch* antibiotics was also the most commonly prescribed regimen for malaria cases in DRC. Conversely, in Kenya and Uganda, *access* antibiotics were more frequently prescribed across the different syndromes, although there was an increase in the prescription of *watch* antibiotics for urogenital syndromes in Uganda and malaria in Kenya. In the latter, this refers to the single malaria case out of 5 that received an antibiotic prescription (combination of both *access* and *watch* antibiotics). *Watch* antibiotics were the preferred option for urogenital syndromes in all three countries (Supplementary Table 2) and country-specific differences in antibiotic choice were most pronounced for undifferentiated syndromes (excluding malaria) where *watch* antibiotics prevailed in the DRC, *access* antibiotics in Uganda, and no antibiotic use in Kenya. The two most frequently prescribed agents for each diagnosis category are presented in Table 3.

**Table 3:** Most frequently prescribed antibiotics, stratified by main initial working diagnosis, in the Democratic Republic of the Congo, Kenya and Uganda, 2021–2024.

Working diagnosis	DRC	Kenya	Uganda
Malaria	Ciprofloxacin	Amoxicillin/Amoxiclav	Amoxicillin/Amoxiclav
	Cefixime	Azithromycin	Ceftriaxone
Undifferentiated Systemic syndrome	Ciprofloxacin	Amoxicillin/Amoxiclav	Ciprofloxacin
	Cefixime	Cefuroxime	Amoxicillin/Amoxiclav
Respiratory Syndrome	Amoxicillin/Amoxiclav	Amoxicillin/Amoxiclav	Amoxicillin/Amoxiclav
	Azithromycin	Azithromycin	Ciprofloxacin
Gastrointestinal Syndrome	Ciprofloxacin	Metronidazole	Metronidazole
	Metronidazole	Ciprofloxacin	Ciprofloxacin
Urogenital Syndrome	Cefixime	Ciprofloxacin	Ciprofloxacin
	Amoxicillin/Amoxiclav	Doxycycline	Metronidazole

Amoxiclav, Amoxicillin/Clavulanic acid; DRC, Democratic Republic of the Congo; Colors: green = access antibiotic; yellow = watch antibiotic (WHO AWaRe Category).

To better understand the drivers of *watch* antibiotic prescription in undifferentiated illness, we performed multivariable logistic regression. A positive Widal test and seeking treatment at a hospital at the time of study enrolment were identified as significant independent predictors with aORs of 37.86 (95% CI 21.01–72.61) and 5.56 (95% CI 2.74–12.31), respectively. Being admitted after enrolment, and presenting with fever and general symptoms, showed more modest associations with aORs around 2.0 (Table 4 and Supplementary Table 4).

## 4 Discussion

Over a period of two-years (October 2021–February 2024), 4,806 patients were enrolled in our multicenter study of subjects attending outpatient consultations for febrile illness, at six health facilities, at various levels in the health system, in East Africa (eastern DRC, Kenya and Uganda). Overall, more than half of the enrolled population presented with non-specific symptoms, including headache, arthralgia and fatigue, with a most prevalent initial working diagnosis of undifferentiated febrile illness in 37.5% of cases. Despite a major lack of a confirmed aetiological diagnosis at enrolment, 72.9% of patients were prescribed antibiotics, including *watch* antibiotics in 32.9% of cases.

It is worth mentioning that, in the absence of more detailed biochemical and microbiological parameters, of patient final/definitive diagnosis and outcome and of the clinical reasoning behind therapeutic choices, it was not possible to assess the adequacy of antibiotic therapy in our study. Furthermore, we did not include subjects under the age of 10, thus limiting results for pediatric patients, whereas adult participants were asked to recall antibiotic use prior to enrolment, which may have resulted in recall bias or underreporting if the antibiotic category of a drug was unknown. A shortcoming of our study was the use of a cross-sectional design: only enrolment visit was used from a longitudinal study, because the follow-up visit of the parent study was 4–6 weeks after initial enrolment, and did not gather information concerning antibiotic use. Adding a follow-up of patients, in a cohort design, 1 week after enrolment to evaluate treatment success or failure, would provide an additional, but limited, information to such a study. On the other hand, our study enrolled patients over 2 years in several countries in the same region and from different healthcare facilities. The strength lies in the coordination among the countries and the use of standardized protocols for data collection, which allowed for comparison and identification of both common patterns and local specificities.

Prior to enrolment, seeking care at the community level was particularly common in DRC and Uganda. It is likely that the dispensary that enrolled participants in Kenya may have been the first point of contact, unlike hospitals and health centers in the other two countries, where patients attending at a later stage, when clinical conditions worsened. Nevertheless, the discrepancy between the prevalent use of pharmacy-based care in DRC versus the use of health centers in Uganda and Kenya stood out. This is in line with a previous study in DRC, where malaria care-seeking individuals relied primarily on drugs originating from the informal sector (self-medication, pharmaceutical store agents, street sellers of drugs, and traditional healers) during the pre-hospital stage (26). Also in Ghana the private retail sector was observed as the first option in care-seeking behavior, even for the poorest (10). Patients’ decisions about where and when to seek care are undoubtedly complex and may depend, among others, on distance from the health facility, disease severity, and direct and indirect costs, especially if health insurance is limited, as in our study sites.

In all three countries, participants sought care in the first week of fever with a fairly non-specific triad of symptoms, including headache, fatigue, and arthromyalgia. However, bleeding, among typical symptoms of RVF (parent study focus), were more frequently described in Uganda, where an outbreak of RVF was occurring during part of our study period (27), while rhinorrhoea and respiratory symptoms were more prevalent in the Kenyan study population. In Kenya, indeed, respiratory disease was the main reason for seeking care, predominantly upper respiratory tract infections in 90.7% of the overall respiratory cases. This is in contrast with a previous household-survey in Kenya in 2018, which reported generally low access to outpatient care for mild respiratory illness (37–43%), substantial levels of self-medication (18–26%), and limited hospitalization for severe pneumonia (18–24%) (28). It should be noted, however, that Kenya experienced multiple COVID-19 waves during our study period (29) and ranked among the top 20 African countries with the highest number of COVID-19 infections (30). In our study, undifferentiated febrile illness (excluding malaria) accounted for approximately 30–35% of cases in Kenya and DRC, and approximately 45% in Uganda. This finding was consistent with 20–50% of undifferentiated fever in children over 5 years of age and adults in Asia and Africa (4), and on the lower end of a recent estimate of 64% from a systematic review in East-African adolescents and adults (1). In the study from Verani *et al*., performing a multiplex PCR for several viral, bacterial, and protozoal fever-causing pathogens detection in Kenya, no pathogens were identified in 54.4% of cases (8). This highlights how challenging the diagnosis and management of febrile illnesses can be, not only in a resource-restricted setting such as that of our study sites, but also when diagnostic tools are available. Relying exclusively on symptoms and provisional diagnoses may result in a substantial proportion of prescriptions being empirical, as has been reported in many African countries (31).

When investigating antibiotic use in the community prior to enrolment, we observed significantly higher rates in the study population from DRC (50.0%) compared to Kenya and Uganda (3.3 and 11.2%, respectively), primarily correlated with seeking care at pharmacies (DRC, Kenya and Uganda) or health facilities (Kenya and Uganda). These findings align with the substantial heterogeneity documented in previous studies. In rural areas of Burkina Faso, outpatient antibiotic use occurred more frequently following health center visits (54.8%) than after visits to pharmacies (26.2%) or informal medicine sellers (26.9%) (32). Similarly, a previous study conducted in DRC reported that over 50% of community-level antibiotic use, mostly from the *watch* antibiotic group, resulted from visits to private healthcare providers, with only 3% attributable to hospital visits (33). Additionally, prior research documented antibiotic use prior to seeking medical care in patients with persistent fever as less than 10% in DRC (34), while a multicountry study including Kenya and Uganda found rates of 36% (35). The substantial variability across these studies highlights the challenges of direct comparison and underscores the importance of considering both between-country and within-country contextual differences when interpreting patterns of antibiotic use.

At the time of study enrolment, antibiotic prescriptions showed higher rates in DRC (87.0%) than in Kenya and Uganda (68.9 and 65.8%, respectively). This difference may in part be explained by DRC enrolling patients exclusively at hospital level, where more severe cases are expected, compared to Kenya and Uganda, which recruited across different levels of health facilities. Nevertheless, looking at the breakdown of the total number of prescribed antibiotics based on the WHO AWaRe classification (19), only Kenya and Uganda reached the target of at least 60% of antibiotic consumption in the *access* group; a threshold set for indirect indication of the appropriateness of antibiotic use which, however, pertains to total antibiotic consumption at the national level (19). This was not the case in the DRC, where only 37.8% of prescribed antibiotics belonged to the *access* group, suggesting a possible overuse of *watch* antibiotics, which could be a concern for proper antibiotic use and the risk of AMR. Urogenital syndromes accounted for the highest use of antibiotics in DRC and Uganda (>90%) and the highest overall use of *watch* antibiotics. This, together with the use of ciprofloxacin as first choice (in Kenya and Uganda), was in line with WHO recommendations on the treatment of urinary tract infections (19). The highest overall use of *access* antibiotics was recorded in respiratory syndromes, with fairly consistent treatment choices across countries, with amoxicillin-clavulanic acid as the first choice. However, given the higher prevalence of upper respiratory tract infections, particularly in Kenya (90.7% of all respiratory infections), this finding contrasted with WHO recommendations, that do not recommend routine antibiotic treatment for most upper respiratory tract infections, unless a bacterial cause is confirmed or strongly suspected (19). Similarly to our study, a previous analysis of antibiotic prescription surveillance in primary care in Kenya, detected nearly 80% of acute respiratory infections (with or without concomitant diagnoses) treated with antibiotics, exceeding national and international recommended prescription levels (36), partly attributed to high doctors’ workloads, perceptions among doctors and patients that doctors should prescribe antibiotics, and, in some cases, the desire of clinics to dispose of expiring drugs (36). In our study, the antibiotic prescription persisted high (at least 80%) and fairly consistent across countries also for gastroenteric syndromes, with ciprofloxacin and metronidazole as first choices, although the WHO indicates the antibiotic as not needed in the majority of watery diarrhoea cases (19). The most substantial differences in antibiotic prescribing were found in the malaria and undifferentiated febrile illness, with undifferentiated fever specifically not being addressed in the WHO AWaRe book. In DRC, antibiotics, particularly *watch* antibiotics, were prescribed in almost all malaria cases, in contrast to Kenya and Uganda. Comparable data were reported in Tanzania, where co-prescription of antibiotics and antimalarials occurred in 20% of cases, predominantly among children under 5 (37). In another study from Burkina Faso and DRC, 40% of malaria cases received *watch* antibiotics, which was attributed to severe malaria as a risk factor for invasive non-typhi *Salmonella* infection, responsible for most bloodstream infections in both countries (11). This may have played a role in our findings of DRC as well, where a positive Widal test was the main factor associated with *watch* antibiotic prescribing. In the DRC study population, almost all cases classified as undifferentiated febrile illness were suspected cases of enteric fever. Despite the many controversies regarding the Widal test usefulness in diagnosing enteric fever in endemic settings (38–42)–being not reliable method for diagnosing acute disease -, this test remains widely used in low- and middle-income countries. The consequences of an incorrect diagnosis of enteric fever are, among others, possible misdiagnosis, antibiotic over-treatment with probable side effects and increased antibiotic resistance, and increased costs (38). Typhoid fever nevertheless remains a poverty-related disease with a high morbidity burden in low- and middle-income countries (43), especially in DRC and Madagascar, with incidence rates being more than 100 cases per 100000 person-years (44). Moreover, *Salmonella* spp. have consistently been classified as high-priority antibiotic-resistant pathogens in the WHO Bacterial Priority Pathogens Lists (45, 46). Non-typhoidal and typhi *Salmonella* which are the most frequently isolated pathogens in bloodstream infections in DRC (respectively 66 and 10% of isolates), have shown an increasing worrying resistance pattern (33, 44) which could also partially explain the higher/systematic use of *watch* antibiotics in our study, with ciprofloxacin and cefixime among the most commonly used. Valia *et al*. reached a similar conclusion, reporting high use of *watch* antibiotics, especially ceftriaxone, when bloodstream infection is suspected without microbiological confirmation in rural Burkina Faso, possibly attributed to the increasing ineffectiveness of ampicillin against *Enterobacterales*.

Antimicrobial resistance is a complex and multidimensional problem (47). While antibiotics are genuinely life-saving when appropriately used, prescribing decisions are influenced by a spectrum of contextual factors ranging from beliefs (48) that “antibiotic is the highest standard of care for all fevers”, to legitimate clinical concerns related to diagnostic uncertainty and limited opportunities for patient follow-up (49). Considering that a significant proportion of our study population (37.5%) was diagnosed with undifferentiated febrile illness, for which no aetiological diagnosis was available, enhancing the local availability of appropriate diagnostic tests (e.g., blood cultures and antimicrobial susceptibility testing) and those suitable for point-of-care use, including the use of biomarkers (e.g., C-reactive Protein and procalcitonin), would better define true indications for antibiotic therapy, and support more targeted and appropriate prescribing (50). Although the potential utility of these biomarkers in primary care centers in SSA has been investigated, with preliminary promising findings coming mainly from a few countries in East Africa, scientific evidence remains limited, with a clear need to fill this knowledge gap in order to inform practice and wider implementation (12, 51). Within a broader policy and health systems framework, NAPs on AMR provide the strategic foundation for stewardship efforts; however, they differ substantially across countries in their scope, priorities, and implementation. Kenya and Uganda are both on their second NAP, while DRC is operationalizing its first (52–55). Elton *et al*. (56) found that AMR Preparedness Scores across SSA were on average 53% lower than the overall Joint External Evaluation (JJE) mean score, with East Africa - Kenya and Uganda - reporting the strongest regional AMR response, while DRC, classified as having no capacity in the JEE assessment. Moreover, DRC is absent from the published evidence evaluating AMR governance and stewardship in the region (47–49, 57–59), highlighting the challenge this poses, further deepened by ongoing economic crises and conflicts (60).

In conclusion, our study described the management of patients with acute febrile illnesses who presented for consultation at six enrolling health facilities in eastern DRC, Kenya, and Uganda. Different patterns of care access, of importance of undifferentiated febrile illness and of use of antibiotics prior to enrolment (particularly in DRC) and at enrolment were observed in the three countries. DRC accounted for the largest share of *watch* antibiotic use (62.2%), possibly related to a high suspicion of enteric fever with resistant strains. Relying solely on symptoms and provisional diagnoses implies that antibiotic prescription is empirical and likely to increase the risk of inappropriate antibiotic use, raising concerns for potential AMR. To strengthen antimicrobial stewardship, NAPs on AMR must be based on interventions tailored to each country’s context, underpinned by dedicated funding, and periodically evaluated through structured progress reports, with due consideration for contextual appropriateness and scalability. In this setting, priority should be given to improving access to diagnostics and point-of-care biomarkers, training healthcare providers in managing diagnostic uncertainty and providing rational antibiotic use guidance for undifferentiated fever, alongside the strengthening of tangible regulations on antimicrobial use that balance stewardship with continued access to medications (48). These efforts must encompass both the formal and informal healthcare sectors, ensuring that stewardship interventions reach all levels of care.

## Data Availability

The datasets presented in this article are not readily available because of privacy reasons, as it contains sensitive patient information. Requests to access the datasets should be directed to ITM’s contact point for data access: ITMresearchdataaccess@itg.be. All requests will be reviewed for approval by ITMs Data Access Committee.
