# Supplementary material for: Patterns of antibiotic use for acute febrile illness in resource-limited settings: a multicenter study in DR Congo, Kenya and Uganda
Source: Front Public Health. 2026 May 29;14:1837179. doi: 10.3389/fpubh.2026.1837179 (PMC13260630; doi:10.3389/fpubh.2026.1837179)
Supplement: Supplementary file 1 [file Supplementary_file_1.pdf]

Supplementary Material

Supplementary Table 1. Sociodemographic and pre-enrolment characteristics of enrolled patients, by main initial working diagnosis, in the Democratic Republic of the Congo (n=1370), Kenya (n=1468) and Uganda (n=1968), 2021-2024.

	DRC					KENYA					UGANDA				
**Variable**	**Malaria n 231**	**Undiff n 472**	**Respir n 217**	**Gastro n 264**	**Urogen n 219**	**Malaria n 5**	**Undiff n 460**	**Respir n 779**	**Gastro n 215**	**Urogen n 12**	**Malaria n 318**	**Undiff n 870**	**Respir n 440**	**Gastro n 205**	**Urogen n 149**
**Occupation**															
HCW	20 (8.7)	45 (9.5)	16 (7.4)	11 (4.2)	6 (2.7)	0 (0)	10 (2.2)	5 (0.6)	1 (0.5)	0 (0)	12 (3.8)	39 (4.5)	13 (2.9)	1 (0.5)	1 (0.7)
Skilled	33 (14.3)	57 (12.1)	21 (9.7)	31 (11.7)	26 (11.9)	3 (60.0)	47 (10.2)	77 (9.9)	16 (7.4)	0 (0)	20 (6.3)	52 (6.0)	30 (6.8)	13 (6.3)	11 (7.4)
Unskilled	36 (15.6)	85 (18.0)	33 (15.2)	51 (19.3)	29 (13.2)	1 (20.0)	104 (22.6)	203 (26.1)	55 (25.6)	1 (8.3)	20 (6.3)	79 (9.1)	23 (5.2)	17 (8.3)	14 (9.4)
Farmer	13 (4.8)	13 (2.7)	10 (4.6)	10 (3.8)	8 (3.6)	1 (20.0)	180 (39.0)	178 (22.8)	81 (37.7)	10 (83.3)	204 (64.1)	548 (63.0)	279 (63.4)	140 (68.3)	108 (72.5)
None	90 (39.0)	188 (39.9)	99 (45.6)	110 (41.7)	115 (52.5)	0 (0)	74 (16.1)	228 (29.3)	36 (16.7)	1 (8.3)	16 (5.0)	52 (6.0)	24 (5.4)	8 (3.9)	3 (2.0)
Student	41 (17.7)	84 (17.9)	38 (17.5)	51 (19.3)	35 (16.0)	0 (0)	45 (9.8)	88 (11.3)	26 (12.1)	0 (0)	46 (14.5)	100 (11.5)	71 (16.4)	26 (12.7)	12 (8.0)
**Education** Low schooling	126 (54.5)	216 (45.8)	111 (51.1)	134 (50.8)	95 (43.4)	3 (60.0)	312 (67.8)	447 (57.4)	146 (67.9)	10 (83.3)	252 (79.2)	679 (78.0)	288 (65.4)	147 (71.7)	112 (75.2)
**Before enrolment**															
Seeking care	97 (42.0)	181 (38.3)	80 (36.9)	80 (30.3)	72 (32.9)	3 (60.0)	33 (7.2)	23 (2.9)	11 (5.1)	0 (0)	100 (31.4)	282 (32.4)	86 (19.5)	47 (22.9)	46 (30.9)
Hospital	8 (3.5)	11 (2.3)	1 (0.5)	5 (1.9)	3 (1.4)	3 (60)	30 (6.5)	16 (2.0)	9 (4.2)	0 (0)	86 (27.0)	257 (29.5)	71 (16.1)	41 (20.0)	38 (25.5)
Traditional healer	1 (0.4)	14 (3.0)	7 (3.2)	10 (3.8)	7 (3.2)	0 (0)	0 (0)	0 (0)	0 (0)	0 (0)	2 (0.6)	5 (0.6)	2 (0.4)	0 (0)	1 (0.7)
Chemist/Pharmacy	83 (35.9)	149 (31.6)	70 (32.3)	65 (24.6)	64 (29.2)	0 (0)	3 (0.6)	7 (0.9)	2 (0.9)	0 (0)	12 (3.8)	20 (2.3)	14 (3.2)	7 (3.4)	6 (4.0)
Friends/family	26 (11.3)	51 (10.8)	13 (6.0)	10 (3.8)	17 (7.8)	0 (0)	0 (0)	0 (0)	0 (0)	0 (0)	2 (0.6)	6 (0.7)	0 (0)	0 (0)	1 (0.7)
Prior antibiotic use	91 (39.4)	234 (49.6)	143 (65.9)	109 (41.3)	105 (47.9)	1 (20.0)	20 (4.3)	20 (2.6)	6 (2.8)	0 (0)	25 (7.9)	93 (10.7)	43 (9.8)	24 (11.7)	28 (18.8)
Prior traditional care	14 (6.1)	43 (9.1)	23 (10.6)	25 (9.5)	16 (7.3)	0 (0)	7 (1.5)	19 (2.4)	5 (2.3)	0 (0)	62 (19.5)	261 (30.0)	99 (22.5)	53 (25.8)	46 (30.9)
Prior admission	17 (7.4)	14 (3.0)	5 (2.3)	6 (2.3)	1 (0.5)	0 (0)	0 (0)	1 (0.1)	0 (0)	0 (0)	20 (6.3)	32 (3.7)	5 (1.1)	6 (2.9)	3 (2.0)

Legend: DRC, Democratic Republic of the Congo; Gastro, gastrointestinal; HCW, healthcare workers; n, number; Respir, respiratory; Undiff, undifferentiated; Urogen, urogenital.

Supplementary Table 2. Distribution of prescriptions after enrolment stratified by main initial working diagnosis, in the Democratic Republic of the Congo, Kenya and Uganda, 2021-2024 (% of participants per working diagnosis).

	DRC					KENYA					UGANDA				
**Variable**	**Malaria** n 231	**Undiff** n 472	**Resp** n 217	**Gastr** n 264	**Urogen** n 219	**Malaria** n 5	**Undiff** n 460	**Resp** n 779	**Gastr** n 215	**Urogen** n 12	**Malaria** n 318	**Undiff** n 870	**Resp** n 440	**Gastr** n 205	**Urogen** n 149
**Prescription**															
Antipyretic	213 (92.2)	436 (92.4)	193 (88.9)	245 (92.8)	207 (94.5)	5 (100)	351 (76.3)	752 (96.5)	159 (73.9)	9 (75.0)	308 (96.9)	751 (86.3)	413 (93.9)	191 (93.2)	137 (91.9)
Antimalarial	228 (98.7)	3 (0.6)	7 (3.2)	17 (6.4)	8 (3.6)	5 (100)	1 (0.2)	0 (0)	1 (0.5)	0 (0)	309 (97.2)	3 (0.3)	20 (4.5)	5 (2.4)	10 (6.7)
Antibiotic	185 (80.1)	420 (89.0)	196 (90.3)	211 (79.9)	214 (97.7)	1 (20.0)	185 (40.2)	642 (82.4)	184 (85.6)	9 (75.0)	61 (19.2)	611 (70.2)	329 (74.8)	180 (87.8)	138 (92.6)
Antibiotic (>1)	15 (6.5)	29 (6.1)	15 (6.9)	69 (26.1)	49 (22.4)	1 (20.0)	13 (2.8)	14 (1.8)	43 (20.0)	3 (25.0)	6 (1.9)	165 (19.0)	43 (9.8)	86 (41.9)	84 (56.4)
Access	29 (12.5)	80 (16.9)	110 (50.7)	56 (21.2)	56 (25.6)	0 (0)	129 (28.0)	542 (69.6)	135 (62.8)	6 (50.0)	43 (13.5)	353 (40.6)	249 (56.6)	89 (43.4)	30 (20.1)
Watch	156 (67.5)	340 (72.0)	86 (39.6)	155 (58.7)	158 (72.1)	1 (20.0)	56 (12.2)	100 (12.8)	49 (22.8)	3 (25.0)	18 (5.7)	258 (29.7)	80 (18.2)	91 (44.4)	108 (72.5)

Legend: DRC, Democratic Republic of the Congo; Gastro, gastrointestinal; n, number; Resp, respiratory; Undiff, undifferentiated; Urogen, urogenital.

Supplementary Table 3. Multivariable analysis of reported antibiotic use before enrolment, in the Democratic Republic of the Congo (n=1359), Kenya (n=1416) and Uganda (n=1878), 2021-2024.

	DRC				KENYA				UGANDA			
**Variable**	**cOR**	**95% CI**	**aOR**	**95% CI**	**cOR**	**95% CI**	**aOR**	**95% CI**	**cOR**	**95% CI**	**aOR**	**95% CI**
**Gender**												
Male	1.02	0.81-1.28			0.58	0.31-1.04			0.99	0.74-1.32		
**Age group**												
10-20 yo												
21-40 yo	0.99	0.74-1.33			2.08	0.69-8.98			1.57	1.05-2.41	**1.81**	**1.06-3.15**
Above 40	0.97	0.70-1.35			4.66	1.63-19.61			1.91	1.22-3.04	**2.10**	**1.15-3.91**
**Education**												
Low level	1.19	0.96-1.47			1.54	0.83-2.98			0.72	0.53-0.99		
**Occupation**												
Farmer												
HCW	2.15	1.05-4.59	**2.44**	**1.12-5.52**	*NA*				2.49	1.29-4.52	**3.06**	**1.41-6.44**
None	3.27	1.77-6.36	**3.35**	**1.72-6.92**					0.37	0.11-0.90	0.61	0.17-1.71
Skilled	3.11	1.60-6.34	**3.43**	**1.66-7.46**					2.62	1.63-4.11	**3.74**	**2.09-6.61**
Student	2.58	1.36-5.16	**2.91**	**1.45-6.17**					1.03	0.64-1.59	**1.98**	**1.07-3.62**
Unskilled	2.16	1.13-4.34	2.01	0.99-4.27					1.11	0.62-1.88	1.31	0.67-2.43
**Trad medicine**												
Yes	5.93	3.74-9.85	**6.16**	**3.66-10.96**	5.09	1.45-13.87			3.54	2.64-4.76	2.10	1.48-2.97
**Prior admission**												
Yes	2.81	1.44-5.91	**3.22**	**1.49-7.47**	*NA*				3.25	1.81-5.62		
**Reported fever**												
Yes	1.06	0.53-2.14			0.64	0.189-4.01			1.17	0.71-2.08		
**Seek care**												
Hospital	1.62	0.74-3.71			128.65	62.94-276.28	**240.50**	**104.92-614.35**	15.04	10.78-21.32	**15.5**	**10.73-22.82**
Traditional	3.45	1.69-7.76			*NA*				3.46	0.74-12.54		
Pharmacy/shop	1.75	1.38-2.21	**2.14**	**1.64-2.82**	58.22	16.92-229.66	**293.34**	**74.78-1330.71**	2.01	0.97-3.80	**5.11**	**2.25-10.81**
Family/friends	0.14	0.08-0.23	**0.08**	**0.04-0.14**	*NA*				2.01	0.30-8.07		
**Symptoms**												
General	1.52	1.19-1.94			1.73	0.92-3.49			1.26	0.83-2.00		
CNS	2.21	1.46-3.41	**2.20**	**1.42-3.49**	0.94	0.51-1.83			0.75	0.47-1.23		
Gastrointestinal	1.31	1.05-1.62	**1.27**	**1.01-1.61**	2.62	1.47-4.80			1.66	1.19-2.34		
Urogenital	1.15	0.87-1.52			4.88	1.39-13.26			1.78	1.27-2.47		
Respiratory	1.18	0.94-1.49			1.12	0.62-2.07			1.01	0.75-1.34		
Muscoskeletal	1.23	0.99-1.53			1.35	0.76-2.42			0.94	0.68-1.31		

Legend: aOR, adjusted Odds Ratio; CNS, Central Nervous System; cOR, crude Odds Ratio; DRC, Democratic Republic of the Congo; HCW, healthcare worker; NA, Not Applicable; Trad, medicine; yo, years old; 95% CI, 95% Confidence Interval.

Supplementary Table 4. Multivariable analysis of *watch* antibiotic prescribing for undifferentiated febrile illness, at enrolment, in the Democratic Republic of the Congo (n=472), Kenya (n=460) and Uganda (n=870), 2021-2024.

**Variable**		**cOR (95% CI)**	**p-value**	**aOR (95% CI)**	**p-value**
Country	Kenya	ref	<0.001	ref	
	**Uganda**	3.04 (2.23-4.20)		1.77 (1.14-2.78)	**0.012**
	DRC	18.58 (13.26-26.43)		1.30 (0.81-2.12)	0.3
Gender	Male	0.95 (0.78-1.15)	0.6		
Age group	10-20 yo		0.009		
	21-40 yo	1.43 (1.09-1.87)			
	Above 40	1.10 (0.83-1.47)			
Occupation	Farmer		<0.001		
	HCW	2.36 (1.52-3.65)			
	None	2.77 (2.11-3.66)			
	Skilled	2.50 (1.75-3.57)			
	Student	2.30 (1.69-3.14)			
	Unskilled	1.66 (1.23-2.23)			
**Enrolling facility**	Dispensary	ref	<0.001	ref	
	Health center	9.13 (4.98-18.77)		1.92 (0.86-4.58)	0.12
	**Hospital**	34.73 (19.22-70.74)		5.56 (2.74-12.31)	**<0.001**
Prior trad medicine	Yes	1.13 (0.87-1.45)	0.4		
Prior admission	Yes	0.61 (0.30-1.16)	0.15		
Reported fever	No		<0.001		
	Unknown	0.31 (0.02-1.66)			
	Yes	3.03 (1.90-5.09)			
**Fever***	Yes	3.12 (2.52-3.87)	<0.001	1.84 (1.39-2.44)	**<0.001**
**Symptoms**	**general**	2.01 (1.58-2.59)	<0.001	1.96 (1.32-2.95)	**0.001**
	CNS	3.49 (2.47-5.05)	<0.001		
	ENT	0.73 (0.56-0.94)	0.02		
	gastrointestinal	1.63 (1.34-1.99)	<0.001		
	**urogenital**	1.32 (0.99-1.75)	0.055	1.69 (1.21-2.37)	**0.002**
	respiratory	1.05 (0.86-1.27)	0.6		
	**muscokeletal**	1.48 (1.20-1.82)	<0.001	1.52 (1.12-2.06)	**0.007**
Malaria positive (≥ 1 test)	Yes	0.88 (0.04-9.18)	0.9		
**Widal positive**	Yes	53.11 (32.29-94.40)	<0.001	37.86 (21.01-72.61)	<0.001
**Admission**	Yes	3.17 (2.19-4.66)	<0.001	2.52 (1.62-3.95)	<0.001

Legend: aOR, adjusted Odds Ratio; CNS, Central Nervous System; cOR, crude Odds Ratio; DRC, Democratic Republic of the Congo; ENT, Ear, Nose and Throat; HCW, healthcare worker; n, number; ref, reference; yo, years old; * fever at enrolment, 95% CI, 95% Confidence Interval.
